# Performance of Thailand’s universal health coverage scheme: Evaluating the effectiveness of annual public hearings

**DOI:** 10.1111/hex.13142

**Published:** 2020-10-09

**Authors:** Kanang Kantamaturapoj, Aniqa I. Marshall, Somtanuek Chotchoungchatchai, Kamonwan Kiewnin, Walaiporn Patcharanarumol, Viroj Tangcharoensathien

**Affiliations:** ^1^ Faculty of Social Sciences and Humanities Mahidol University Nakhon Pathom Thailand; ^2^ International Health Policy Program Ministry of Public Health Nonthaburi Thailand

**Keywords:** National Health Security Act, policy decision, public hearing, Thailand, Universal Health Coverage Scheme

## Abstract

**Background:**

Legislative provisions in Thailand's National Health Security Act 2002 mandate annual public hearings for providers, beneficiaries and other stakeholders in order to improve the performance of the Universal Health Coverage Scheme (UCS).

**Objective:**

This study aims to explore the annual public hearing process, evaluate its effectiveness and propose recommendations for improvement.

**Method:**

In‐depth interviews were conducted with 29 key informants from various stakeholder groups involved in annual public hearings.

**Results:**

The evaluation showed that the public hearings fully met the criteria of influence over policy decision and partially met the criteria of appropriate participation approach and social learning. However, there are rooms for improvement on public hearing's inclusiveness and representativeness of participants, adequacy of information and transparency.

**Conclusions:**

Three recommendations were proposed a) informing stakeholders in advance of the agenda and hearing process to enable their active participation; b) identifying experienced facilitators to navigate the discussions across stakeholders with different or conflicting interests, in order to reach consensus and prioritize recommendations; and c) communicating policy and management responses as a result of public hearings to all stakeholders in a timely manner.

## 1 INTRODUCTION

Thailand achieved Universal Health Coverage (UHC) following the adoption of the National Health Security Act (NHSA) in November 2002, which led to the implementation of the Universal Health Coverage Scheme (UCS), the largest public insurance scheme covering approximately 75% of Thailand's 68 million population.^1^, ^2^, ^3^, ^4^, ^5^ The NHSA mandates the National Health Security Office (NHSO) to implement the UCS through its head quarter and 13 regional offices.^6^ The NHSO functions as a strategic purchaser of health services and is responsible for ensuring accountability to UCS members.^7^ Various studies have confirmed that the implementation of UCS leads to equitable access to health services, a low level of unmet health needs and a high level of financial risk protection.^2^, ^4^, ^5^, ^8^, ^9^

Public participation in policy decision making is a precondition of deliberative and participatory democracy; an essential prerequisite for people acceptance of public policies.^10^, ^11^ Engaging the public in health policy decisions enhances health systems’ responsiveness to people's need.^12^ The legislative process in 2001 endorsed public participation provisions in the NHSA as proposed by civil society organization (CSO) representatives. Five members from nine CSO constituencies 1) children and adolescents, 2) women, 3) elderly, 4) disabled or mental health patients, 5) HIV or other chronic disease patients, 6) labour, 7) slum communities, 8) agriculturists and 9) ethnic minorities were appointed by the Cabinet to serve in the Governing Board of NHSO.^13^ The NHSO also translates the legislative intention into practice by opening the space for public participation in various dimensions such as amendments of the benefits package, enhancing rights protection, improving responsive health service provisions and ensuring adequate funding for health promotion and protection.^7^

Annual public hearings are a platform through which the public can participate in improving UCS performance. As mandated by Article 18 (13) of the NHSA, the National Health Security Board (NHSB) shall conduct annual public hearings for service providers (including doctors, nurses and other public health personnel) and UCS beneficiaries (including patients).^13^ These public hearings have been conducted annually since 2004, resulting in enhancements in the UCS benefits package which meet the needs and rights protection of its members.^14^ These include the establishment of the Rehabilitation Fund in 2004, authorization for UCS members to re‐register with new health‐care provider networks up to four rounds a year since 2012 until now and expansion of maternity health services of previously limited to two pregnancies per woman to more than two pregnancies in 2015.^15^

Although, the NHSO has conducted public hearings for over 15 years, an evaluation of the public hearing process and outcomes has never been conducted and the impact of public hearings on UCS policy decision making remains unclear. This study aims to explore the annual public hearing process, to evaluate its effectiveness against six criteria drawn from literatures and to propose recommendations for improvement.

## 2 THEORETICAL FRAMEWORK

Meaningful participation refers to effective construction and implementation of participation as well as public acceptance of public participation outcomes.^16^ Approaches to evaluating meaningful participation depends on the specific issues under consideration and related contexts ^17^; therefore, there is no single accepted evaluative framework applicable to all cases.^17^, ^18^ This study investigated evaluation frameworks for meaningful participation from various scholars ^10^, ^16^, ^19^, ^20^, ^21^ and selected 6 evaluation criteria that fit the context of annual public hearings of health providers and UCS beneficiaries. These are (a) inclusiveness and representativeness, (b) adequacy of information, (c) appropriate participation approach, (d) social learning, (e) transparency and (f) influence over policy decision making. The details on the evaluation criteria, definition and indicators are presented in Table 1.

**Table 1:** Evaluation criteria for meaningful participation of annual public hearing

Evaluation criteria	Definition	Indicator
1) Inclusiveness and representativeness of participants	Both direct stakeholders ^16^, ^24^ and wider public ^10^ are involved in the public participation.	1) Potential affected stakeholders by the new proposals of UCS attend the annual public hearing. Interested lay public attend the annual public hearing.
2) Adequacy of information	Adequate information is provided to the participants to be able to seek for assistance,^27^ to strengthen their understanding and to be able to participate actively.^26^	1) The public hearing organizers disseminate necessary information related to the agenda well in advance through various channels to the potential participants. 2) The public hearing organizers assist the participants to prepare the information to discuss in public hearing.
Appropriate participation approach	Observations of contexts, culture, norms, timing, and diversity of participants are considered ^17^, ^22^, ^27^ as key attributes of appropriate public participation approaches.^28^, ^29^	The approach and process of the public hearing is flexible and appropriate which fit to the context, culture and norms of the community. 2) All groups of stakeholders participating feel comfortable with public hearing approach.
Social learning	Participants listen to each other and find a solution which accommodates mutual interests.^30^, ^31^, ^32^, ^33^, ^34^ The experienced facilitators support and empower participants to express their opinions in a constructive manner.^32^	1) The public hearing is held in a neutral place. 2) All stakeholders are free to express opinions in a friendly environment and work together to reach the conclusion on common interests and reconcile the differences. 3) The facilitators have extensive experience in moderating public hearing sessions.
Transparency	The outcomes of the public participation reflects the discussions, conclusions and decisions of participants and can be justified..^10^, ^20^	1) The participants clearly understand how the issues of public hearing are framed. 2) The mechanism to reach mutual agreements of all participants has been jointly developed by all participants.
Influence	The outcome of public participation process influenced over policy decision making.^16^	1) The participants are informed that their opinions are put into policy decision‐making process. 2) The policy decision which reflects the public hearing outcomes are published and publicly available.

## 3 METHOD

Using the evaluation framework in Table 1, this study assessed public hearings at regional and national levels. One province per region (Northern, North‐Eastern, Central and Southern) was randomly selected as the study site. As national level public hearings are conducted in Bangkok, the public hearings in Bangkok were additionally evaluated. In total, five study sites were selected for the evaluation.

Data were collected through in‐depth interviews with key informants, chosen from a list of previous annual public hearing participants. In addition to executives from the NHSO, at least one key informant from each stakeholder group was selected: public hearing organizers, health‐care providers, Civil Society Organization (CSO) members and local administrative organization members (Table 2).

**Table 2:** the number of key informants by group and site

Key Informant Groups	National	Northern	North Eastern	Central	Southern	Total
NHSO Executives	2					**2**
Public hearing organizers	2	1	1	1	3	**8**
Health‐care providers	1	1	4	1	1	**8**
Civil Society Organizations	1	1	1	1	1	**5**
Local administrative organizations	1	2	1	1	1	**6**
**Total**	**7**	**5**	**7**	**4**	**6**	**29**

Ethics approval was obtained from the Institute for the Development of Human Research Protection, COA No. IHRP2019095 (dated 28 October 2019). Informed consent was obtained and interviews with 29 key informants were carried out between October and December 2019. Each interview was recorded, transcribed and encoded to maintain the privacy of the key informants.

A coding scheme was developed to categorize and formulate characteristics and themes from the interviews. The scheme aimed to address four key issue areas: (1) How stakeholders are involved in the annual public hearings, (2) What information is provided to the stakeholders, (3) What participation methods are used in public hearing activities, and (4) What barriers and constraints impede the implementation of the public hearings.

Six criteria for meaningful participation (Table 1) were used as the framework for the evaluation. Data were mapped into the framework matrix to analyse whether the public hearing process met each criteria. Based on the findings from interviews and data triangulation, achievement was measured into three levels: fully meeting the criteria (the finding supports all indicators), partially meeting the criteria (the finding supports some indicators) or not meeting the criteria (the finding do not support any indicators).

## 4 RESULTS AND DISCUSSION

### 4.1 The process of annual public hearings

The guiding principles of the public hearings and appointment of the ad‐hoc committee for proposing annual public hearing agendas are endorsed by the NHSB (NHSO3).

In 2019, the NHSB approved 8 issues to be addressed at the annual public hearing. Seven issues are ‘cross cutting issues for all regions’, which include the following: (a) type and scope of health services, (b) service standards, (c) NHSO management, (d) national health security fund management, (e) local health fund management, (f) public participation, and (g) perception and rights protection. The 8th issue area in the public hearing agenda is local or context specific (NHSO1,2,3; NHSR2,3,6; CSO4).

The public hearings begin at the regional level, with regional forums at each of the 13 NHSO regions, followed by a national forum in Bangkok. The regional forums are organized by the NHSO regional health security offices that are responsible for selecting the number and arrangement of each forum to the suit local situation (NHSO2,3; NHSR2,6).

In the Central and North Eastern regions, the regional health security offices assign working groups, comprised of representatives from health‐care providers, CSOs and local administrative organizations, to organize the regional forums (NHSR2,6). For provincial forums, in the Central region, the working group assigns the provincial CSO that coordinates the UCS to organize the provincial hearings; each province then nominates a representative to the regional forum. Whereas in the North Eastern region, the working group assigns local universities to organize the provincial forums, the discussions from the provincial forums then inform the regional public hearings (NHSR2; PRO4,6; CSO3; LAO3). In contrast, both provincial and regional level public hearings in the Northern and Southern regions are organized and managed by office staff of the regional health security offices (NHSR#6, CSO2; LAO1,2).

Recommendations and feedback based on the forum discussions at the regional public hearings are compiled and submitted to the Regional Health Security Board and the Regional Standard and Quality Control Board (NHSR3, CSO4 and PRO7). In addition, according to the public hearing organizers, the Regional Health Security Board and the Regional Standard and Quality Control Board have co‐hosted regional public hearings since 2017, allowing both boards to provide immediate response to key issues discussed at the hearings (NHSO3). Confirmed by all key informant groups, challenges that can be addressed at the regional level are taken into consideration by the regional boards and not forwarded to the national level public hearings (NHSR2,3,4,5,6; PRO2; CSO2,4; LAO1,5). Similarly, the provincial level challenges and recommendations are not referred to the regional hearings and are instead addressed provincially (NHSR2,3,5,6; CSO4). This streamlines the bureaucratic inertia of addressing major concerns.

The recommendations from the regional public hearings to be considered at the national level are compiled by NHSO to inform the national public hearing. The national forum commences with a presentation on previous year's hearing including related policy and management responses to avoid redundancy and duplication of problems already considered or addressed (NHSO1,2,3). The proposals from the 13 regional public hearings are then presented, with participants able to make additional recommendations that have not already been covered (NHSO2,3). The recommendations endorsed at the national public hearings are collated by the ad‐hoc committee and handed to the NHSB for further deliberation and action. The NHSB appoints related committees to then implement any approved proposals and disseminates a public hearing report to inform on the progress of the proposals, including issues that were addressed, proposals not implemented, and recommendations that require further research or not within NHSBs responsibility and need referrals to relevant agencies (NHSO,1,2,3,4).

### 4.2 The evaluation of annual public hearings for health‐care providers and UCS beneficiaries

Using the evaluation framework proposed in Table 1, this study sought to identify current gaps and areas where the public hearings can be improved.^22^, ^23^

#### 4.2.1 Inclusiveness and representativeness

Literature suggests that partners in public participation should include individuals, groups or organizations that may be affected by the policy decision.^16^, ^24^ Participation by the wider public is crucial in ensuring decisions incorporate the views and values of affected populations.^10^ Although, allowing everyone with different interests and expectations to participate can be challenging due to cost implications and administrative complexities, the engagement of diverse stakeholders can reduce the possibility of overlooking certain challenges faced by different stakeholders.^16^, ^22^, ^25^ Thus, both direct stakeholders and interested lay public should be involved in the public hearings.

This study found that the stakeholders that may be affected by any changes to the UCS are represented in the annual public hearings, and this includes UCS beneficiaries, health‐care providers and local administrative organization. However, public hearings are not open to all interested lay public and only invited participants are eligible to participate as the NHSO is required to pay for the transportation costs for all attendees (NHSO 3,4). Regarding the recruitment of participants at public hearings, the beneficiary representatives are selected by CSOs, and representatives of providers are selected by the Provincial Health Office, while local administrative organizations select participants from their pool of members. Although participants are nominated, the representatives truly able to provide constructive opinions to the UCS system are not always chosen or do not attend the public hearings. For example, technical level staff working in the hospital's UHC centre are always nominated and attended while top level hospital managers rarely attended the public hearings due to other obligations (PRO8,9). As a result, the opinions from the health‐care provider representatives mostly cover problems at the operational level where solutions do not often address the root cause which requires a policy action.

‘Top level managers are extremely busy. They rarely attend public hearing. The doctors do not waste their time to attend public hearing as well. They have appointment with patients. The queuing in hospital is very stressful and these should be solved by hospital managers not technical level officers’. (PRO9)

The informants from local administrative organizations in the Northern region reported that the invitation letters are always received at the very last minute (LAO1,2). Additionally, the organizers do not follow‐up on the invitation letters; therefore, their representatives miss the opportunity to attend (LAO2,3).

‘I received the invitation letter on the day of public hearing. I live in another province which takes 2 hours drive to the public hearing venue. The organizer never call us first to inform about the public hearing. If they call, we can prepare to come. I am a member of NHSO line group but they did not communicate about the meeting in the line’. (LAO2)

The interview found that the public hearing organizers in the Central region use their personal relationship to invite participants they knew to ensure representation in the forum (NHSR3; PRO7; CSO4; LAO4). Good relationships between public hearing organizers and stakeholders can encourage participation in the public hearing more so than official invitation letters. However, the downside is diminished participant diversity as the same persons are invited every year due to established relationships with the organizers (PRO 4,5,6,7; CSO5).

Due to low diversity of individual participants and lack of involvement from more groups and wider organizations, the participants involved in the annual public hearing cannot meet the criteria of inclusiveness and representativeness.

#### 4.2.2 ADEQUACY OF INFORMATION

Information provision is crucial in ensuring meaningful participation. It is important that participants receive adequate information prior to attending public hearings to be able to strengthen their understanding and actively participate in the process.^16^, ^26^ It is suggested that experts should help participants prepare evidence and presentations for proposals.^27^ The public hearing organizers should not only disseminate necessary information related to the agenda but should also assist participants in preparing information to discuss during the forums.

This study found that public hearing organizers and other stakeholders have differing views on the adequacy of information provided. The organizers believe that the information provided on each agenda item and the implementation of previous recommendations to minimize redundancy in the discussions is sufficient for all participants (NHSO1,2,3; NHSR2,4,6). However, most participants reported that the information was not distributed in advance of the public hearing. In the North Eastern region, documents are provided on the day of the hearing, and organizers do not inform participants on the process of the public hearing or how participants should prepare in order to make relevant contributions to the forum (PRO4,5,6,7). Similarly, in the Southern region, the organizers do not notify participants on the process of the public hearing, and as the process changes yearly, participants often face difficulties in preparing proposals and make recommendations (CSO5).

‘I don’t know how I should prepare my proposals. I need to know about the process and the issues on the agenda. I want to propose suggestions that are useful for UCS improvement’ (PRO6)

Although organizers do not provide assistance in preparing participants for the public hearing, this study found that CSOs groups solicit opinions among themselves to improve UCS through their UHC network (CSO1,2,3,4,5). The CSO network collaboratively propose and provide evidence to voice their opinions in all public hearing forums to increase their influence over relevant issues (CSO1).

Due to lack of information provision and absence of stakeholder preparation, improvements are still necessary for the public hearings to meet the criteria of adequacy of information.

#### 4.2.3 APPROPRIATE PARTICIPATION APPROACH

The best public participation method should take into account the local contexts, culture, social norms in the community and timing and characteristics of stakeholders.^17^, ^22^, ^27^ The organizers should select participation methods where all groups of stakeholders can equally express their opinions and avoid neglecting minorities^28^, ^29^ Thus, the approach and process of the public hearing should be flexible and appropriate to the local context. Additionally, all groups of stakeholders should feel comfortable with the selected public participation approach.

This study found that the NHSO allows each region to manage public hearings to suit their local needs (NHSO3). In the North Eastern, Central and Southern regions, the provincial level hearings are conducted prior to the regional hearings. In contrast, in the Northern region, only one public hearing at the regional level is conducted. However, the public hearing approaches are based on the ability of organizers instead of the preferences of stakeholders. Key informants from all stakeholder groups agreed that only one approach to the public hearings is insufficient and alternative methods should be explored such as website surveys, questionnaires or mobile applications (NHSO2,3; NHSR4,5,6; PRO5,8; CSO4; LAO1,2).

In addition, some informants reported that they were not comfortable in expressing their opinions at the regional or national forums due to the formality of the process (NHSR3; LAO1,2,4; CSO3). Additionally, key informants from the provider group reported that they did not feel free to express true opinions or their needs in front of beneficiaries due to fear of leading to conflict (PRO4,5,6,7).

‘Small meetings are better because participants feel comfortable expressing their opinions. Small provincial level meetings should be organized because the true voice of people can be heard’. (LAO4)

‘We are the defendants in front of the UCS beneficiaries in these public hearings. We have to explain about our limitation to the beneficiaries when they complain about the hospital services. We don’t have time to propose what we want. Also, expressing our heavy work loads due to new UCS package in front of the UCS beneficiaries can lead to conflict’. (PRO5)

This study found that the annual public hearing forums are flexible across regions based on the ability of regional organizers. However, some participants have reported being uncomfortable in expressing their opinions in such formal settings or when confronted with potential opponents. Therefore, the annual public hearings only partially meet the criteria of appropriate participation approach.

#### 4.2.4 Social learning

Public participation supports social learning among members of society, where participants listen to each other and try to find solutions which accommodate mutual interests.^30^, ^31^, ^32^, ^33^, ^34^ According to the literature, location and facilitators are key factors in stimulating social learning in public participation. Public hearings must be located in a neutral setting in order to enable social learning^35^ and should be conducted by neutral facilitators.^36^ In addition, the ability and experience of facilitators are important in supporting and empowering participants to express their opinions in a constructive manner.^32^ To evaluate social learning of public hearings in the Thai UCS, three indicators are considered. Firstly, whether the public hearing is held in a neutral place. Secondly, whether all stakeholders are free to express opinions in a friendly environment and work together to reach the mutual conclusion. Thirdly, whether the facilitator has extensive experience in moderating public hearing forums.

The result from interview found that public hearings in all the study sites are carried out in hotels where stakeholders could easily attend. In most cases, the forums for health‐care providers are separate from the beneficiaries as the stakeholders have different interests and to avoid confrontation and potential conflicts (NHSO1,3; NHSR4). Such separation into two groups prevents participants from learning about the perspectives of other stakeholders who may have different views and opposing positions. In the North Eastern region, key informants from the provider group reported that in forums attended by a mix of providers and beneficiaries, a lot of time is spent on providing explanations to the beneficiaries (PRO4,5,6,7). One key informant from the provider group stated that he was willing to hear the perspectives of beneficiaries towards UCS, as the public hearings provided a space for all stakeholders to voice and listen to each other (PRO1).

‘When the beneficiaries complained about UCS in the hospital, we must explain our limitations. We hope the beneficiaries listen and understand us more’. (PRO6)

Key informants from NHSO and the organizers explained that the public hearings also enable learning between NHSO and stakeholders. When stakeholders make recommendations that can not be implemented, facilitators have the opportunity to explain the limitations faced by NHSO. As a result, the stakeholders are able to better understand UCS (NHSO2,3; NHSR3,4) and the responsibility of NHSO (NHSO1).

‘The public hearing is a learning platform between NHSO and stakeholders. We learn what stakeholders think and whether they understand us. If they understand us, they would make proposals within the mandates of NHSO’. (NHSR4)

Key informants from beneficiary and local administrative organization groups found that too much time is spent on presentations (LAO1, CSO2), which results in limited time for discussions to find solutions (CSO2). In addition, the key informants indicated that the facilitators are not able to create a learning environment (LAO1) and are unable to encourage all groups to express their true needs (PRO7). The facilitators spend too much time emphasizing ‘cooperation’ between providers and beneficiaries; therefore, messages from providers’ view are not shared to prevent disruption of cooperative efforts (PRO4,5,6,7). Similarly, key informants from beneficiaries feel uncomfortable expressing their real needs when facilitators stress the importance of compromise and reconciliation (with health‐care providers) (CSO2).

‘They used a process of compromise but the time was very limited. They closed the hearing session at noon. There was no time to submit my proposal’. (PRO5)

The study found that the annual public hearings enable social learning in terms of neutral location which facilitates listening to other stakeholders’ opinion. However, an adequate leaning environment and experienced facilitators are lacking. Thus, the annual public hearings in Thai UCS only partially meet the criteria of social learning.

#### 4.2.5 Transparency

Transparency in public participation refers to the openness in framing issues and the mutual decision‐making mechanisms among stakeholders.^10^, ^20^ The public participation process should be transparent, so the public can see how decisions are being made.^16^ In order to measure transparency of annual public hearings, two indicators were considered. Firstly, whether the organizers explained how the issues of public hearings are framed. Secondly, whether the organizers developed and utilized mechanisms to attain mutual agreements on decision‐making processes among participants.

For the public hearings, the NHSO released seven issues as a framework for the hearings. However, many key informants did not understand why these seven issue areas in the public hearing agenda were selected, and found that the issue areas were too general (PRO8) and not aligned with participants interests (PRO7,9; NHSR5). In the participants’ perspective, the issues were not clearly framed, and therefore, participants are unsure whether their issues fit with agenda items (PRO5,8).

‘My concern was not related to the seven issues on the agenda. I would like to propose about primary health care, but I was not sure which agenda I should propose’ (PRO8)

This study found that the organizers only compile opinions from participants, with no mechanism to attain mutual agreements on decision making among participants. Key informant from the provider group felt their proposals were not fully captured as the organizers had already pre‐prepared a conclusion (PRO2).

The public hearing organizer key informants indicated that the NHSO regional offices were responsive by immediately taking key actions as per the recommendations suggested by the participants (NHSR2,3,4,5,6). However, this was countered by the key informants from both provider and beneficiary groups that believed the organizers (NHSO regional offices) only complied issues without taking action, as there were many recommendations suggested that were not prioritized due to lack of mechanism for consensus and prioritization of issues of importance across stakeholders (PRO9; CSO2,5).

‘We only go and voice our concerns. There is no process of consensus or agreement’. (PRO9)

As there were no explanations to the public on the reasonings behind the selection of seven issues in the public hearings and no mechanisms to achieve a consensus on the various proposals, the annual public hearings in Thai UCS do not meet the criteria of transparency.

#### 4.2.6 Influence over policy decision making

The public hearing organizers should inform the stakeholders and general public on how the public participation process influence policy decision making.^16^, ^37^ The perception of stakeholders that their contributions influence policy decision making bolsters stakeholders’ enthusiasm for involvement in future participation.^38^ In order to assess the influence of public hearings over the policy decision making, two indicators were used. Firstly, whether the participants are informed on the process by which their opinions are considered by policy makers. Secondly, whether the policy decisions reflecting the public hearing outcomes are published and publicly available.

This study found that the organizers always inform the participants that the output of the public hearings will be considered by the National Health Security Board. New benefit packages derived from public hearing discussions of the previous year are presented in the subsequent annual public hearing. In addition, the NHSO disseminates an annual report on the public hearings to disclose the number of proposals recommended at the forums, the number of proposals which were fully implemented and the process of implementation (NHSO2,3).

However, many key informants found the reports to be too general, and not a reflection of the recommendations and opinions expressed at the public hearings (NHSR5; PRO7,9; LAO4). As participants are unable to identify their proposed issues, most were uninterested in reading the full public hearing reports (NHSR3,5; PRO5,8; CSO4,5; LAO4). Additionally, some key informants did not read the reports as they were not aware a public hearing report had been developed (PRO4,6). The local administrative organizations explained that it takes too long for the public hearing reports to be disseminated (LAO#3).

‘The public hearing report was an overview of issues. It did not respond to my proposed issues’. (PRO7)

Due to the fact that the participants are subsequently informed on how their opinions and recommendations are considered through the publication of publicly available annual public hearing reports, the annual public hearings fully meet the criteria of influence on policy decision making.

## 5 CONCLUSION AND RECOMMENDATIONS

This study evaluated the annual public hearings for the improvement of UCS performance by applying a six criteria evaluation framework. The evaluation was based on the experiences and opinions of 29 key informants from various stakeholder groups and analysed through categorizing the information into the pre‐determined evaluation criteria.

The evaluation found that the public hearings fully meet the criteria of influence over the policy decision making as several key policy reforms have been generated from public hearing findings. For example, access to and payment of emergency health services either at public or private hospitals were harmonized across the three public health insurance schemes in 2012, the criteria for no‐fault financial assistance were revised in 2013, and the two‐child limit on the number of birth deliveries eligible for the UCS was abolished in 2015.^7^

The public hearings partially meet the criteria of appropriate participation approach and social learning. However, the public hearings have a deficiency in meeting the three other criteria of inclusiveness and representativeness of participants due to low diversity of participants and lack of involvement from wider public; adequacy of information due to lack of information provision and absence of stakeholders’ preparation; and transparency due to lack of capacity to reach consensus on the proposals across different stakeholders by the moderator of the public hearings.

There are some limitations to this study. Firstly, due to budget constraints, the authors could only randomly select 5 out of the 13 NHSO regions as study sites; therefore, the results may not be nationally representative. Secondly, the evaluation was dependent on either positive or negative experiences of key informants which may introduce biases towards certain issues or organizations.

The strength of the annual public hearing is that it is mandatory by law through the NHSA. The public hearings not only result in policy and management responses to improve the UCS performance, but also serve as a key platform for health‐care providers and beneficiaries to meet and resolve common problems through interactive discussions from different perspectives, actors and interests in a constructive manner.

Three recommendations emerged from this study. Firstly, the study found that the NHSO allows its regional offices to organize the public hearing, which has been gradually adapted to suit the local context, culture and norms of each region. However, the public hearing organizers should inform the stakeholders in advance about the agenda and process of the public hearing, and how to prepare their proposals and recommendations. Participating in complex issue like UCS requires the support from organizers to prepare proposals. We agree with Sinclair and Diduck^39^ that empowering participants should be done such as providing funding support and access to experts who can assist in understanding the system and prepare for active participation is important.

Secondly, this study found that public hearing organizers only compile the issues from participants, with no mechanisms to reach consensus on which proposal is accepted and is prioritized for further actions. Therefore, the public hearing organizers should identify experienced facilitators to navigate the discussions across stakeholders with different interests and concerns. The facilitators should support deliberative discussions among stakeholders, especially health‐care providers and beneficiaries that often have different opinions and insist on their own perspectives, to reach a consensus and agree on issues that are to be prioritized. This study found that approaches which push too much towards compromise does not support social learning. We agree with Schusler, Decker and Pfeffer^40^ that the moderator should urge participants to bring up the conflicting points of view in order to identify shared values and common solutions which require further deliberation in the future.

Lastly, the study found that the NHSB fully used the findings and recommendations from public hearings to improve UCS performance; however, the lack of effective communication to stakeholders is a major gap. Influence of public hearings over decisions has positive effect on acceptability by all concerned parties.^41^ Therefore, the NHSO should establish feedback mechanisms for timely reporting of policy and management responses to all concerned stakeholders and share them to the wider public beyond the individual, group or organizations that have attend the public hearings.

## CONFLICT OF INTEREST

Authors declare that they have no conflict of interest.

## Data Availability

Data sharing is not applicable to this article as no new data were created or analysed in this study.
